# Characterization, sources, and risk assessment of PAHs in borehole water from the vicinity of an unlined dumpsite in Awka, Nigeria

**DOI:** 10.1038/s41598-023-36691-3

**Published:** 2023-06-15

**Authors:** Chiedozie Chukwuemeka Aralu, Patrice-Anthony C. Okoye, Hillary O. Abugu, Nkiruka C. Eboagu, Victor Chukwuemeka Eze

**Affiliations:** 1 Department of Pure and Industrial Chemistry, Nnamdi Azikiwe University, Awka, Nigeria; 2 Department of Pure and Industrial Chemistry, University of Nigeria, Nsukka, Nigeria; 3 Department of Chemistry, University of Agriculture and Environmental Sciences, Umuagwo, Imo, Nigeria

**Keywords:** Environmental sciences, Chemistry, Analytical chemistry, Environmental chemistry

## Abstract

Polycyclic aromatic hydrocarbons (PAHs) are contaminants of interest in the ecosystem due to associated health risks. Therefore, their detection in the environment is important. In this regard, the risk assessment of PAHs in borehole water near the unlined dumpsite in Anambra State was investigated. Samples of borehole water (16 each) were collected from the study and control areas during both seasons. The PAH concentrations in the borehole water samples were analyzed using gas chromatography. The mean PAH concentration in the study and control samples for the wet season varied from BL–7.65 µg/L to BL–2.98 µg/L, respectively. The study samples' dry season values ranged from BL to 3.33 µg/L, while control samples ranged from BL to 1.87 µg/L. $\sum$PAHs for the wet and dry seasons varied from 5.8 to 13.94 µg/L and 4.25 to 10.09 µg/L for study and control samples, respectively. The four and five rings PAH were the most dominant group in the $\sum$ PAHs for the study and control samples, respectively. Diagnostic ratios suggested pyrolytic and petrogenic sources for both locations. The cluster analysis showed different sources of the congeners in the samples. The non-carcinogenic risk showed no possibility of risks via dermal and ingestion routes. In addition, the possibility of cancer risks via ingestion routes was doubtful. The carcinogenic risk index through dermal contact exceeded the acceptable limit for adults and is at a tolerable limit for children, indicating potential threats to humans, with adults more susceptible to cancer risks. Therefore, this study recommends that sanitary dumpsites be constructed for waste disposal and implementation of environmental laws to prevent underground water pollution and the environment.

## Introduction

Accompanied by industrial expansion, rapid urbanization, and swift economic development, water pollution has become a serious environmental threat in Nigeria^1–3^. Anambra State has witnessed significant economic growth and development, which has increased waste output. Poor waste management system associated with unlined dumpsites has been a menace to the environment due to the percolation of leachates^4–7^. These leachates contain organic and inorganic pollutants, which damage the ecosystem if not properly treated before discharging. Leachates from domestic/agricultural wastes and discharge of untreated effluents are various ways of PAH pollution in the environment^8–12^. PAHs are ubiquitous organic constituents formed during the combustion processes of biomass, fossil fuel, garbage, and industrial activities^6,13,14^.

PAHs are organic compounds that have attracted global recognition because of their carcinogenic threats^15,16^. PAHs are categorized into high (4–6 rings) and low-member (2–3 rings) weight groups^17–19^. PAHs are generally classified as relatively persistent organic and environmental pollutants^20–22^. Higher molecular weight PAHs groups are relatively immovable, and moderately insoluble in water^18,23^. PAHs are normally found in the bottom sediments, thus accumulating to greater concentrations, which can be lethal to the environment^24^.

Certain PAHs occur at low environmental concentrations due to their low biodegradability and elimination problems^18–27^. Also, the PAHs have bioeffects, such as interactive effects on hematological parameters and developmental toxicity^28,29^.

Underground/surface water can be polluted with PAHs via leaching from landfills, petroleum spills, and fossil fuel combustion, which have attracted global attention^30–32^. Populace living around waste Sites can be exposed to PAHs through pollution of the borehole water via leachates^17,20,33^. In the current study, the Agu-Akwa dumpsite in Awka was considered. The dump site is an open/unlined dumpsite, the most common method of waste disposal in Nigeria due to poor budgetary allocation^34–36^. Contamination of the underground water, such as boreholes, is expected since the refuse dump is highly unregulated due to the release of toxic pollutants.

Previous studies have been done on the contamination effects of leachates on underground water^37–40^. Most work has focused mainly on groundwater's physicochemical and heavy metals contamination. Aralu et al*.*^7^ investigated the pollution effect of PAHs in the Nnewi metropolis, Anambra State. However, studies have not been conducted on PAH status in borehole water around the Agu-Awka dump site in the Awka metropolis in Anambra State. The urgent need to investigate the health implications of using the boreholes is very important, considering the proximity of the dumpsite to residential homes in the area.

Therefore, the work was done to determine the PAH concentrations of the boreholes around the dump site. Also, to determine the sources and compositions of the PAHs in the underground water, and to assess the health risks of residents using the borehole water for domestic use. The results can serve as key data in reviewing existing laws on waste management.

## Materials and methods

### Study area

**Figure 1:**
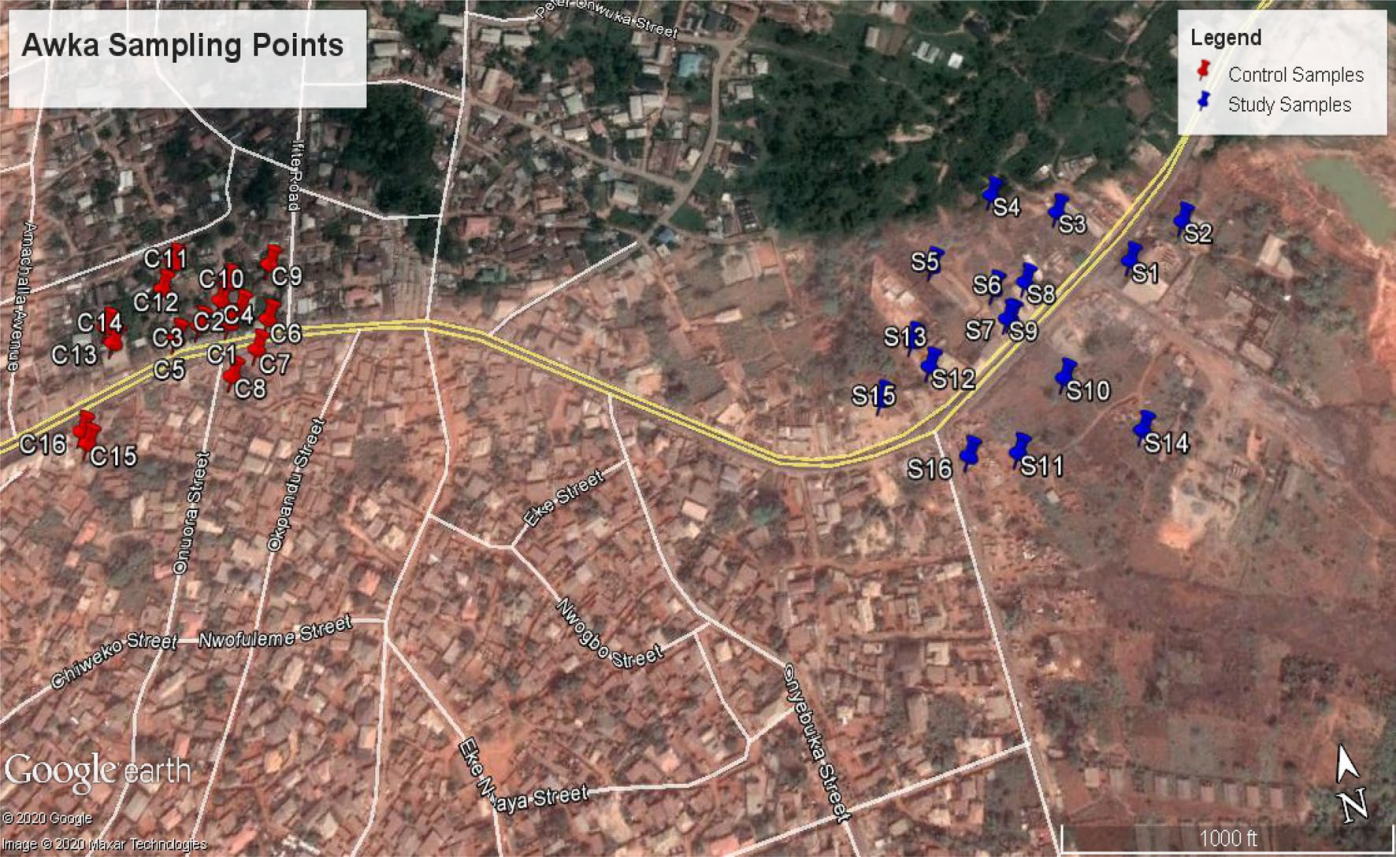
Sampling points description.

Figure 1 and Table 1 show the sampling coordinates and sampling points area within the dump site area. The map in Fig. 1 was generated using Google earthpro version 7.3.1. The sampling points are within the Agu-Awka dumpsite in Awka South, Anambra State. The refuse dump is an unlined/open dumpsite with no preventive liners to prevent leachate. The wastes are openly burnt in the open atmosphere, which releases dangerous fumes. Awka South lies within the tropical rainforest region and Anambra Basin in South-Eastern Nigeria. The city has experienced significant economic development and rapid population growth. The natural vegetation of the area has been affected due to deforestation of the environment due to urbanization. The climate of the location is composed of rainy and dry seasons. The average rainfall varies from 165 to 1025 mm annually. The area's average temperature varies between 27 and 28ºC, which experiences its highest peak at 35 °C^41^. The area's relative humidity varied between 85 and 100% during the wet season and less than 70% during the dry season^41,42^.

**Table 1:** Sampling coordinates.

Sample points			Control points		
Points	Latitude	Longitude	Points	Latitude	Longitude
S1	6.219668	7.091002	C1	6.221057	7.08278
S2	6.219913	7.091621	C2	6.221156	7.082889
S3	6.220296	7.090515	C3	6.221102	7.082509
S4	6.220628	7.08998	C4	6.221284	7.082726
S5	6.220093	7.08921	C5	6.22104	7.082287
S6	6.219728	7.089689	C6	6.221005	7.083127
S7	6.21943	7.089746	C7	6.220756	7.082995
S8	6.219718	7.089989	C8	6.220573	7.082746
S9	6.219429	7.089712	C9	6.221507	7.083206
S10	6.218784	7.090027	C10	6.221424	7.082812
S11	6.218253	7.089396	C11	6.221739	7.082339
S12	6.219187	7.088888	C12	6.221521	7.082213
S13	6.21945	7.08881	C13	6.221142	7.081725
S14	6.21818	7.090541	C14	6.221297	7.081679
S15	6.219003	7.088372	C15	6.220308	7.08142
S16	6.218332	7.088964	C16	6.220425	7.081378

The average elevation above sea level is about 70 m^43^. The soil is characterized by the Imo Shale formation, which consists of blue-grey shale with sand clays, loamy, clay, and sandstones^44^. The Imo Formation is Paleocene in age^40^. The area has a mean depth to the water table of about 16–35 m and a mean static water level of about 40 m^40^. The dump site is located around an industrial area comprising markets, factories, workshops, and construction companies. The types of waste in the dumpsite comprise paper, plastic materials, aluminum, metal objects, batteries, lubricants, and household wastes.

### Sampling and preparation of borehole samples

Sixteen borehole water samples each were collected monthly for the study and control samples, respectively. The wet season was sampled for 4 months (May–August 2018), while the dry season was sampled for 3 months (Dec 2018–February 2019). The study samples were collected within 152–213 m from the dumpsite area, while a distance of 619–788 m away from the study samples was used to collect the control samples. Before sampling, glass sample bottles were washed with detergent, rinsed with distilled water, and dried in an oven. Properly cleaned glass bottles were used to collect borehole water samples. The 16 samples from each location were combined to form a homogenous sample representing the samples collected from a particular location. The homogenized water samples were stored in the refrigerator at 4 $^\circ$C before analysis.

### Chemicals used for the analysis

A standard mixture of 16 US EPA priority PAHs was procured from Accustandards Inc (USA). Analytical grade dichloromethane, acetonitrile, acetone, n-hexane, and anhydrous sodium sulphate were acquired from Sigma-Aldric, USA.

### Preparation of borehole samples and clean-up

The analysis was carried out using the method^45^. 10 mL of the sample was extracted with 200 mL of dichloromethane. The separation of the mixture was carried out using a separating funnel and was concentrated with the aid of a rotary evaporator. The concentrated sample was analyzed by adding 1 mL of acetonitrile. Residue cleaning was performed using an 8 mL (12 mm 5 cm long) glass chromatography column from Restek, USA. The sample was passed through a chromatographic column loaded with 14 g of activated silica gel (60–100 mesh) deposited with glass wool and anhydrous Na_2_SO_4_ (2 g). It was conditioned with 7 mL of n-hexane. The concentrated extract was dissolved in 2 mL n-hexane and loaded into the column. The eluate collected was concentrated using a rotary evaporator. The concentrated eluate was used for analysis after dissolving it with 1 mL of acetone.

### Quantitative analysis of PAHs

A Buck Scientific M910 gas chromatograph (USA) coupled with a flame ionization detector was utilized for the analyses. A column type HP 88 with dimension (100 m × 0.25 μm thickness) CA., USA, and an on-column automatic injector were used for PAH detection. Helium (carrier gas) with a maintained flow rate of 1.5 mL/minand oven ramprates of 6 °C/min was utilised for the experiment. The oven temperature was programmed to start at 70 °C and increase to 300 °C. The detector was operated at 325 °C. The injector temperature was set at 280 °C. The inlet temperature was set at 290 °C. 1 μL was the injected volume using a split mode with a ratio of 5:1^46^.

### Validation of experiment

100 mL of borehole water sample collected from a different location (blank) was spiked with 1 mL standard PAH solution. It was properly extracted using 200 mL of dichloromethane, clean up of the GC column was done, and the concentrated extract analysed for PAHs using the procedure stated by Omores et al*.*^46^. The intraday and interday precision was determined by analyzing the prepared samples on the same day and three different days, respectively. Triplicate analyses were done for the recovery experiment. The overall average recovery rates were 90.6–98.8% (Table 2) and within acceptable limits^18,47^. The limits of detection for the PAHs are also shown in Table 2. The analyte peak was identified by comparison of sample retention time values with those of the standard compounds^48,49^. All calibration curves of the tested PAHs were found to be linear with a correlation coefficient (r^2^ ≥ 0.991) within acceptable limits^18^. The analyses of the samples were performed in triplicate, and the mean results were recorded in Table 3.

**Table 2:** Recovery data and limit of detection for PAHs.

PAHs		Recovery percentage	Limit of detection (μg/L)
Acenaphthene	Acp	90.6 $\pm$ 2.45	0.06
Acenaphthylene	Acy	91.4 $\pm$ 2.89	0.02
Fluoranthene	Fla	90.9 $\pm$ 3.02	0.03
Benzo (k) fluoranthene	BkF	93.5 $\pm$ 3.12	0.18
Benzo (b) fluoranthene	BbF	94.6 $\pm$ 2.15	0.11
Fluorene	Flur	92.8 $\pm$ 1.56	0.02
Benz (a)anthracene	BaA	92.5 $\pm$ 1.48	0.07
Pyrene	Pyr	93.1 $\pm$ 2.25	0.04
Naphthalene	Nap	91.8 $\pm$ 2.13	0.09
Benzo (a) pyrene	BaP	94.7 $\pm$ 3.25	0.03
Dibenz (a,h) anthracene	DbahA	98.8 $\pm$ 1.24	0.05
Phenanthrene	Phen	96.2 $\pm$ 1.47	0.02
Benzo (g,h,i) perylene	BghiP	98.1 $\pm$ 1.17	0.10

**Table 3:** Contents of PAHs in borehole samples.

PAHs (µg/L)	TE ${\mathrm{F}}_{\mathrm{e}}$	ME ${\mathrm{F}}_{\mathrm{e}}$	Wet season				Dry Season			
			Study sample		Control sample		Study sample		Control sample	
			Range	Mean	Range	Mean	Range	Mean	Range	Mean
Acp	0.001	–	BL–0.5	0.25 ± 0.29	BL–3.2	1.03 ± 1.51	BL	–	BL	–
Acy	0.001	–	BL–0.1	0.05 ± 0.06	BL–0.2	0.05 ± 0.1	0.1–0.1	0.1 ± 0.00	BL–0.2	0.67 ± 0.12
Fla	0.001	–	BL–0.2	0.05 ± 0.1	BL–0.3	0.08 ± 0.15	0.2–1.4	0.6 ± 0.69	BL–1.0	0.37 ± 0.55
BkF	0.1	0.11	BL–0.2	0.05 ± 0.1	BL	–	BL–0.2	0.67 ± 0.12	BL	–
BbF	0.1	0.25	BL–7.9	3.13 ± 3.85	0.8–4.8	2.98 ± 2.13	1.1–6.1	3.1 ± 2.65	0.8–3.9	1.87 ± 1.76
Flur	0.001	–	BL–0.3	0.1 ± 0.14	BL–0.1	0.28 ± 0.49	BL	–	BL	–
BaA	0.0	0.082	BL–1.2	0.65 ± 0.55	BL–1.0	0.35 ± 0.47	BL–0.4	0.27 ± 0.23	BL–0.1	0.33 ± 0.06
Pyr	0.001	–	BL–11.8	7.65 ± 5.23	BL	–	BL–6.0	3.33 ± 3.06	BL–0.5	0.17 ± 0.29
Nap	0.001	–	BL–4.4	1.33 ± 2.09	BL–1.3	0.5 ± 0.57	BL–5.3	1.76 ± 3.06	BL–0.3	0.17 ± 0.15
BaP	1	1	BL–0.4	0.1 ± 0.2	BL–0.2	0.05 ± 0.1	BL–0.2	0.13 ± 0.12	0.1–0.1	0.1 ± 0.00
DbahA	1	0.29	BL	–	BL	–	BL–0.1	0.03 ± 0.06	BL	–
Phen	0.001	–	BL–1.7	0.43 ± 0.85	BL–1.8	0.45 ± 0.9	BL	–	BL–1.4	0.47 ± 0.81
BghiP	0.01	0.19	BL–0.6	0.15 ± 0.3	BL–0.1	0.03 ± 0.05	BL–0.2	0.1 ± 0.1	0.1–0.1	0.1 ± 0.00
$\sum \mathrm{PAHs}$	–	–	11.6–17.2	13.94	3.3–7.1	5.8	6.2–13.3	10.09	1.5–6.7	4.25
$\sum$LMW	–	–	0.1–4.5	2.16	1.5–4.2	2.31	0.1–5.4	1.86	BL–1.6	1.31
$\sum$HMW	–	–	8.1–14.2	11.78	1.8–5.1	3.49	6.1–8.9	8.23	1.0–5.1	2.94
$\sum$cPAHs	–	–	1.0–8.1	3.93	1.8–5.0	3.38	1.7–6.4	4.2	0.9–4.0	2.3

*BL* below limit, *LMW* low molecular weights, *HMW* high molecular weights, cPAHs carcinogenic PAHs.

### Health risk assessment

The study calculated health risks using the benzo (a) pyrene toxicity equivalent (${\mathrm{BaP}}_{\mathrm{eq}}$). The ${\mathrm{BaP}}_{\mathrm{eq}}$ was computed using the expression in (Eq. 1)^18,50^.

1 $${\mathrm{BaP}}_{\mathrm{eq}}=\sum {C}_{e}\times {\mathrm{TEF}}_{\mathrm{e}}$$

${C}_{e}$ and ${\mathrm{TEF}}_{\mathrm{e}}$ indicates PAH's concentration and toxicity factors, respectively (Table 3). The health risk was also calculated using the benzo (a) pyrene mutagenic equivalent quotients (${\mathrm{BaP}}_{\mathrm{Meq}}$). The ${\mathrm{BaP}}_{\mathrm{Meq}}$ was computed using (Eq. 2)^51,52^.

2 $${\mathrm{BaP}}_{\mathrm{Meq}}=\sum {\mathrm{C}}_{\mathrm{e}}\times {\mathrm{MEF}}_{\mathrm{e}}$$

${C}_{e}$ and ME ${\mathrm{F}}_{\mathrm{e}}$ indicates the concentration and mutagenic factors of corresponding PAH (Table 3). Health risks were calculated using risk equations for dermal and ingestion pathways^53,54^.

The average daily dosage by dermal interaction (${\mathrm{ADD}}_{\mathrm{dermal}}$) was evaluated for non-carcinogenic risks using (Eq. 3).

3 $${\mathrm{ADD}}_{\mathrm{dermal}}=\frac{\mathrm{C}\times \mathrm{SA}\times \mathrm{KP}\times \mathrm{ET}\times \mathrm{EF}\times \mathrm{ED}\times \mathrm{CF}}{\mathrm{BW}\times \mathrm{AT}}$$

The exposure route by chronic daily intake via ingestion (mg/kg/day) was determined using (Eq. 4) for non-carcinogenic risks.

4 $${\mathrm{CDI}}_{\mathrm{ingestion}}=\frac{\mathrm{C}\times \mathrm{IR}\times \mathrm{EF}\times \mathrm{ED}}{\mathrm{BW}\times \mathrm{AT}}$$

where ${\mathrm{ADD}}_{\mathrm{dermal}}$ corresponds to the average daily dosage by dermal interaction (mg/kg/day); C represents levels of PAHs (mg/L); EF refers to the frequency of exposure (350 days/year); ED refers to the duration of exposure (20 years and 6 years for adult and child respectively)^55^; BW denotes for the body weight (80 kg and 15 kg corresponds to the adult and child weight respectively)^55,56^; AT denotes average life span (7300 days and 2190 days for adult and child respectively)^57^; SA represents the dermal surface area (19,652 cm^2^ and child: 6365 cm^2^ for adult and child respectively)^55^; ET denotes the exposure time of shower and bathing (adult: 0.71 h/day; child: 0.54 h/day)^55^; ${\mathrm{CDI}}_{\mathrm{ingestion}}$ is the chronic daily intake (mg/kg/day); IR stands for the water ingestion rate (adult: 2.5 L/day; child: 0.78 L/day)^55^. The Kp (cm/hr) stands for permeability coefficient (Nap: 0.047; Phen: 0.14; Fla: 0.22; BaA: 0.47; BbF: 0.7; BaP: 0.7; DbahA: 1.50; Pyr: 0.324)^58^; CF represents conversion factor (L/1000 cm)^58,59^.

The HQ and HI, which represent hazard quotient and hazard index, were calculated for individual PAHs using the following equations^60,61^.

5 $$\mathrm{HQ}=\frac{\mathrm{ADD}}{\mathrm{RfD}}$$

6 $$\mathrm{HQ}=\frac{\mathrm{CDI}}{\mathrm{RfD}}$$

7 $$\mathrm{HI}=\sum \mathrm{HQs}$$

RfD stands for dermal reference dose for PAHs (Nap: 0.02; Flur 0.04; Phen: 0.04; Fla: 0.04; Pyr: 0.03 and BghiP: 0.04)^57^. The probability of exposure to a possible carcinogen was evaluated using incremental lifetime cancer risk (ILCR) for carcinogenic PAHs. Lifetime average daily dose (LADD) (mg/kg/day) and lifetime chronic daily intake (LCDI) (mg/kg/day) in (Eqs. 8, 9) was used to evaluate the LADD (dermal contact) and LCDI (ingestion route). The average time (AT) used for ILCR was 25,550 for adults and children. HI was calculated using (Eq. 10)^58^.

8 $$\mathrm{ILCRs}=\mathrm{LADD}\times \mathrm{CSF}$$

9 $$\mathrm{ILCRs}=\mathrm{LCDI}\times \mathrm{CSF}$$

10 $$\mathrm{HI}=\sum \mathrm{ILCRs}$$

CSF stands for the cancer slope factor, which was extrapolated by multiplying the CSF for BaP (7.3 mg/kg/day) by the toxic factor of individual PAHs^53^.

### Statistical estimation

Microsoft Office was used for calculating the mean standard deviations of the sample results. A hierarchical cluster dendrogram was used to assess the relationship between the PAH parameters using OriginPro 9.0. Pearson's correlation analyses at 0.05 significant levels assessed the results between the study areas of the boreholes using SPSS software.

### Ethical approval

All the authors have read, understood, and complied as applicable with the “Ethical responsibilities of Authors” as found in the Instructions for Authors.

## Results and discussion

### Levels of PAHs in the sample

**Figure 2:**
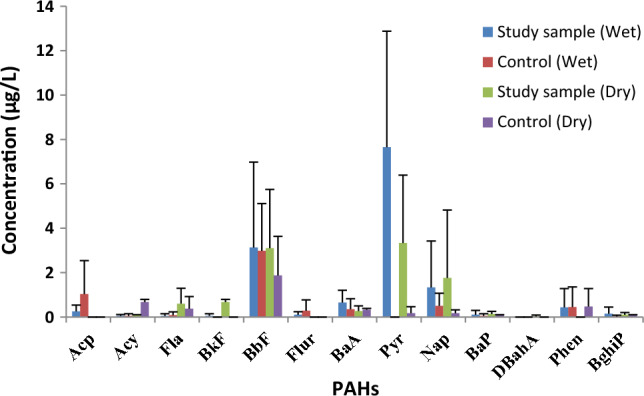
Variation of wet and dry seasons PAH values for both locations.

The mean results for the borehole water samples are illustrated using Table 3. The data in Table 4 shows the comparative study results of the study area with other regions. The borehole samples recorded different PAH concentrations for both locations, confirming the pollutants' ubiquitous nature. Some values were below limit (BL) in the experiment. The wet season PAH mean values varied from BL to 7.65 µg/L for study locations and BL to 2.98 µg/L for control locations. The level of PAHs in the dry season varied from BL to 3.33 µg/L for study locations, while control areas varied from BL to 1.87 µg/L. The wet season values were higher than the dry season, which might be attributed to the leaching of pollutants from the refuse dump and surface runoff through rainfall^62–64^. The mean study sample values (Fig. 2) were higher than the control sample values due to the infiltration of leachates from the dumpsite^12^. The BaP values were lower than the permissible limits of 200 μg/L and 700 μg/L for both locations^59,65^. The values of BaP, which ranged from 1.2 to 4.3 µg/L, were higher than the study sample's values^62^. The values obtained in Tehran, Iran, which ranged from BL to 0.01 μg/L, were lower than the present study^66,67^.

**Table 4:** Comparison of the PAH concentrations (µg/L) from this study with other previous works.

Locations	Range	References
Awka, Nigeria	BL–7.65	This study
Rivers, Nigeria	BL–1.7762	^32^
Nnewi, Nigeria	B–113.13	^12^
Lagos, Nigeria	0.006–2.963	^68^
Akure, Nigeria	BL–0.072	^68^
Rivers, Nigeria	4.25–9.03	^69^
Rivers, Nigeria	BL–3.79	^70^
Rivers, Nigeria	0.13–328.9	^71^
Abia, Nigera	0.51–55.11	^62^
Imo, Nigeria	0.30–42.17	^62^
Chennai, India	BL–143.2	^72^
Taipei, Taiwan	BL–0.0279	^73^
Kaoshiung, Taiwan	0.008–0.33	^73^
Taichung, Taiwan	BL–0.0227	^73^
Rio de Janeiro, Brazil	0.05–84.9	^74^
Jiangsu, China	BL–6.6	^75^
Zhejiang, China	BL–0.05	^76^
Tehran, Iran	0.0324–0.7331	^67^

The low molecular weight PAHs occur mainly in lower concentration values as a result of their high volatility and dissolution^62^. Naphthalene which is a low molecular weight PAH is mainly from petrogenic sources usually from oil spills^77^. It is of importance to note that the values of naphthalene found were within the range of 1.33–1.76 μg/L for the study samples and 0.17–0.5 μg/L for control samples. The relatively high values obtained could be attributed to leaching leachates, oil spills and surface runoff^68,69,77–79^. The values obtained for fluorine varied from BL–0.1 μg/L to BL–0.28 μg/L for study and control samples respectively for both seasons. These values were lower than 0.18–204.38 μg/L obtained in a study conducted by Sun et al*.*^77^.

Higher molecular weight PAHs comprising four or more aromatic rings were also detected in the borehole water samples. Pyrene had the highest concentration (7.65 μg/L) of individual PAHs in the borehole water. BbF is a colourless, aromatic hydrocarbon with five fused rings formed through incomplete combustion of organic matter^51^. The individual PAHs mean values of BbF which ranged from 3.1 to 3.13 μg/L for the study sample and 1.87 to 2.98 μg/L for control samples, were lower than the values reported in a study conducted by Onydinma et al*.*^62^.

DbahA is a five-fused benzene ring produced from the incomplete combustion of organic matter^43^. Worthy of note is that DbahA was the least detected PAH and occurred at a relatively lower concentration in the samples with a range of BL–0.13 μg/L. The values of Fla ranged from 0.05 to 0.6 μg/L and 0.08 to 0.37 μg/L for study and control samples, respectively. The obtained values were lower than the values reported by Edet et al*.*^69^.

Table 4 compares the total PAH levels in borehole water samples located within the dumpsite with borehole samples located in other dumpsites in other regions. High levels of PAHs higher than the study areas were found in Abia and Imo, Nigeria^62^, Rivers, Nigeria^71^, Chennai, India^72^, Rio de Janeiro, Brazil^74^, Tehran, Iran^76^, Nnewi, Nigeria^12^. The PAH levels were similar to those obtained in Jiangsu, China^75^ and Rivers, Nigeria^69^. However, low levels of PAHs lower than the study area results were found in Rivers, Nigeria^31,32,70^, Lagos and Akure, Nigeria^68^, Taipei, Kaoshiung, Taichung regions of Taiwan^73^, Zhejiang, China^76^.

**Figure 3:**
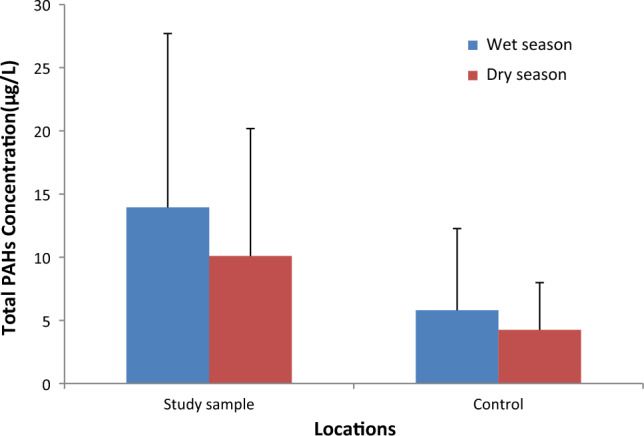
Total PAHs concentration in both seasons.

The PAHs found in the borehole water samples comprised low and high molecular weight PAHs, which was reported previously^7,12,18^. The PAH values revealed that the borehole samples were contaminated with varying PAH concentrations due to their proximity to landfill leachates for both locations, which agrees with previous literature^6,7,12^. The summation level of PAH values $(\sum \mathrm{PAHs})$ obtained in Fig. 3 showed that the study samples had the highest values of 13.94 µg/L and 10.09 µg/L for wet and dry seasons, respectively. The study samples $(\sum \mathrm{PAHs})$ values were greater than the control samples for both seasons, which was attributed to the runoff of leachates from the dumpsite due to its proximity to the study samples^12^.

**Figure 4:**
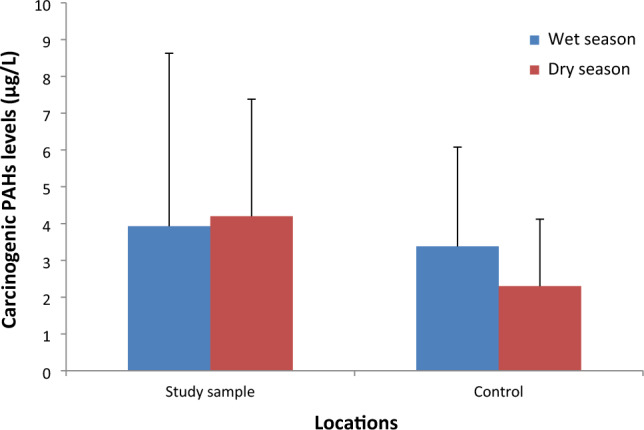
Total carcinogenic PAHs concentration in both seasons.

The wet season $\left(\sum \mathrm{HMW}\right)$ and $(\sum \mathrm{LMW})$ values were greater than the dry season values due to more contamination of the borehole water through leachate runoff^12,80^. The carcinogenic PAHs (cPAHs) levels were evaluated from the borehole water results in Table 3 and Fig. 4. The $\sum$cPAHs in the wet season showed 28.19% for study samples and 58.28% for control samples, while the study and control samples showed 41.63% and 51.11%, correspondingly, during the dry season. The $\sum$cPAHs revealed that the study sample values were greater than the control samples in both seasons. It was attributed to the discharge of leachate from the refuse dump that contributed to the pollution of the borehole samples^12,18^. In the study areas, the levels of PAHs during the wet season followed: Pyr > BbF > Nap > BaA > Phen > Acp > BghiP > Flur, BaP > Acy, Fla, BkF > DBahA, while the control location was BbF > Acp > Nap > Phen > BaA > Flur > Fla > Acy > Bap > BghiP > BkF, Pyr, DBahA. The dry season levels of PAHs obeyed this order for the study site: Pyr > BbF > Nap > BkF > Fla > BaA > BaP > Acy, BghiP > DBahA > Acp, Flur, Phen, while the control site obeyed this order: BbF > Acy > Phen > Fla > BaA > Pyr, Nap > BaP, BghiP > Acp, BkF, Flur, DBahA.

In the wet season (Table 5), the correlation between the study and control locations showed a weak positive correlation (r = 0.229, p = 0.432). The p value (p > 0.05) was non-significant, which implied that the difference between the study and control samples was not statistically significant. In the dry season, the concentration of PAH values has a moderate positive correlation between the study and control sample values (r = 0.535, p = 0.048), which revealed a significant difference between both samples. The correlation values between the study samples (r = 0.880, p = 0.000) and control samples (r = 0.929, p = 0.000) for both seasons showed a significant positive correlation. It indicated that the study and control sample's PAH levels in the wet season were higher in the dry season due to the influx of leachates from the dumpsite.

**Table 5:** Pearson correlation between study area parameters across both seasons.

Correlations	Study sample (wet season)	Control sample (wet season)	Study sample (dry season)	Control sample (dry season)
Study sample (wet season)	1			
Control sample (wet season)	0.229	1		
	0.432			
Study sample (dry season)	0.880**	0.509	1	
	0.000	0.063		
Control sample (dry season)	0.270	0.838**	0.535*	1
	0.351	0.000	0.048	

**Correlation is significant at the 0.01 level (2-tailed).

*Correlation is significant at the 0.05 level (2-tailed).

Consequently, the over-dependence on these boreholes by individuals residing around the dumpsite for a long time may result in several human health conditions.

### Characterisation of ring size

**Figure 5:**
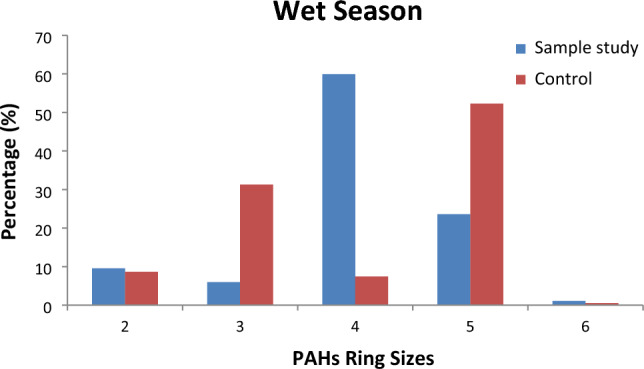
Ring size distribution of PAHs in both locations.

**Figure 6:**
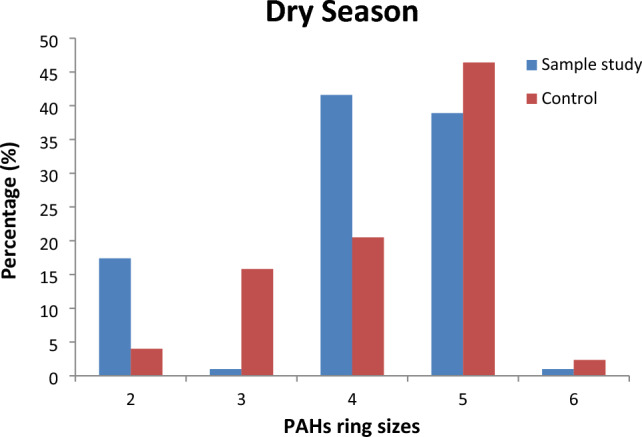
Ring size distribution of PAHs in both locations.

Figures 5 and 6 show the ring size arrangement in the borehole water samples for both locations. The 4-ring PAHs recorded the maximum value (59.92%) during the wet season at the study location, while 52.3% was observed at the 5-ring PAHs. The maximum value of 41.6% was observed during the dry season for 4-ring PAHs, while the 5-ring PAHs recorded a 46.4% maximum value. The 6-ring PAHs recorded the lowest value of 0.52% in the wet season. The ring size profile generally showed that the HMW-PAHs had a higher percentage contribution than the LMW–PAHs. This finding isin agreement with similar studies^12,69,81^ but not in agreement with the study conducted by Aderonke et al*.*^82^ and Adedosu et al*.*^81^ where the LMW–PAHs were the dominant PAHs. The dominant high molecular weight PAHs were attributed to the incomplete combustion of organic materials and solid wastes from the dumpsite^81^. The LMW–PAH's presence in the ring structures is linked to the emission of oil spills and non-combustible matter^81^.

### Source identifications of PAHs

Isomeric ratios of PAH have been applied in the determination of possible input sources of PAH and their transport properties^33,83^. In the present study, PAH source identification was carried out using diagnostic ratios^50^. Fla/Pyr PAHs ratio < 1 implies petrogenic, while > 1 implies pyrolytic^84^. BaA/228 ratio showing < 0.2 suggests petrogenic, while 0.2–0.35 implies pyrolytic sources^85^.

The ratio of ΣLMW/ΣHMW was < 1 for the study and control locations (Table 6), suggesting a dominant pyrolytic source due to incomplete combustion of refuse or biomass^18,85,86^. Also, BaA/228 ratio showed a petrogenic input for both the study sample and control sample locations. The Fla/Pyr diagnostic ratios suggested that study sample locations were from petrogenic sources, while control sample locations confirmed pyrolytic sources. Generally, the PAH contamination in both study areas originated from pyrolytic sources, largely due to incomplete combustion of biomass, discharge of untreated leachates and surface runoff, while the petrogenic sources were due to combustion of petroleum products and oil spills. A predominant petrogenic source was observed in the study sample, while in the control sample, the pyrolytic sources were the dominant PAHs source.

**Table 6:** Isometric ratio values and sources.

PAH ratios	Study sample		Control sample	
	Ratio value	Source	Ratio value	Source
$\sum \mathrm{LMW}$/$\sum \mathrm{HMW}$	0.263	Pyrolytic	0.181	Pyrolytic
BaA/228	0.002	Petrogenic	0.001	Petrogenic
Fla/Pyr	0.059	Petrogenic	2.647	Pyrolytic

### PAHs cluster analysis

**Figure 7:**
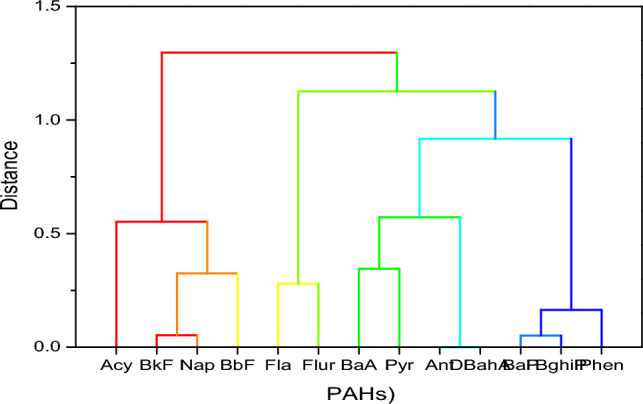
Hierarchical cluster dendrogram for the wet season.

The hierarchical cluster dendrogram (HCD) showed that the PAH congeners in the borehole samples during the wet season were grouped into four clusters (Fig. 7). Acy, BkF, NaP, and BbF are in the first cluster, Fla and Flur in the second cluster, BaA,Pyr, Ant, DBahA, in the third cluster, while BaP, BghiP, and Phen are in the fourth cluster. The first cluster mainly comprises 5, 3, and 2-membered ring PAHs, while the second comprises 5 and 3-membered PAHs. Fluorene, fluoranthene, chrysene, and pyrene are markers for oil combustion^87^. The third and fourth clusters comprised 5, 4, and 3-membered rings and 5, 6, and 3-membered rings, respectively.

**Figure 8:**
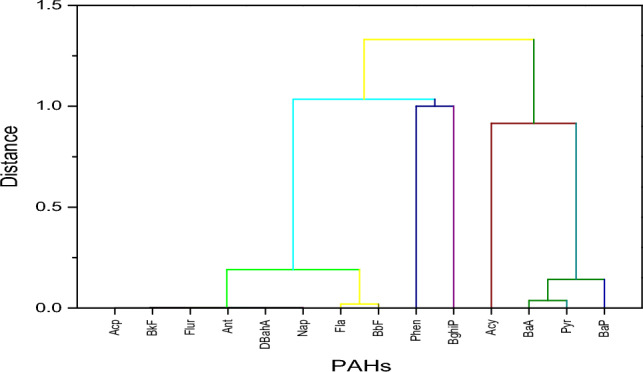
Hierarchical cluster dendrogram for the dry season.

During the dry season, the PAHs were grouped into two main clusters Acy, BaA, Pyr, and BaP are in one cluster, while the rest are in the second cluster (Fig. 8). The first cluster mainly comprises 5, 3, and 4-membered ring PAHs. The second cluster comprises 6,5,4, 3, and 2-membered ring PAHs. The difference in the clustering during the wet and dry seasons could be attributed to the leachate runoff caused by rainfall during the wet season (seasonal variation) and concentrations of the PAHs congeners where most were undetectable during the dry season^6,88^.

### Toxicity and mutagenic equivalent assessment

**Figure 9:**
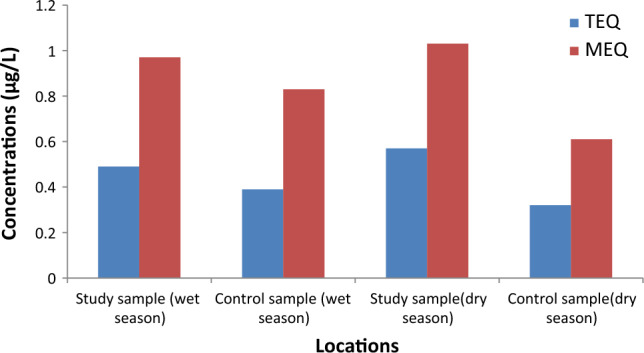
Toxicity and mutagenic equivalent concentration.

The summation of the benzo(a) pyrene toxicity and mutagenic equivalent (TEQs and MEQs) are presented in Fig. 9. The TEQ value for the sample study in the wet season was 0.49, while for the control study was 0.39. The TEQ value for the dry season was 0.57 and 0.32 for the study and control samples, respectively. The MEQ values in the wet season were 0.97 for the study sample and 0.83 for the control sample. The dry season values were 1.03 and 0.61 for the study and control samples, respectively.

The TEQ and MEQ values for the study sample locations were higher than those at the control locations in both seasons, which might be attributed to the infiltration of pollutants from the refuse dump^6,7,12^. The BbF followed by BaA contributed significantly to the TEQ values. The BbF followed byBaP contributed significantly to the MEQ values. The individual PAH's contribution to the TEQ and MEQ could trigger carcinogenic and non-carcinogenic health effects^89^.

### PAHs risk assessment of borehole water samples

Hazard quotient (HQs) values obtained from the average daily dose (${\mathrm{ADD}}_{\mathrm{derm}}$) are shown in Table 7. The HQ and HI values obtained via skin absorption were < 1 for age categories and locations. Therefore, the possibilities of non-carcinogenic risks are very unlikely^21^. The HI values for the child were higher than the adult, which agrees with previous work^62,69,86,90^.

**Table 7:** Hazard quotient values of average dermal dose.

PAHs	${\mathrm{HQs}}_{\mathrm{ Dermal}}$ (ADULT)		${\mathrm{HQs }}_{\mathrm{Dermal}}$ (CHILD	
	Study Sample	Control Sample	Study Sample	Control Sample
Fluoranthene	2.99 $\times {10}^{-4}$	1.7 $\times {10}^{-4}$	3.93 $\times {10}^{-4}$	2.72 $\times {10}^{-4}$
Naphthalene	2.96 $\times {10}^{-4}$	6.4 $\times {10}^{-5}$	3.89 $\times {10}^{-4}$	8.44 $\times {10}^{-5}$
Pyrene	9.92 $\times {10}^{-3}$	1.54 $\times {10}^{-4}$	1.3 $\times {10}^{-2}$	2.02 $\times {10}^{-4}$
HI	1.05 $\times {10}^{-2}$	3.88 $\times {10}^{-4}$	1.38 $\times {10}^{-2}$	5.58 $\times {10}^{-4}$

Cancer risk through dermal exposure is shown in Table 8. ILCR values (1.E−06) are deemed satisfactory, above 1.00E−05 but lesser than 1E−04 are tolerable, while values $\ge$ 1.0 $\times {10}^{-4}$ indicate severe threats^91^. The ILCR values were less than 1 $\times {10}^{-4}.$ The hazard indices showed that the adult HI was predominantly higher than the child HI for both locations, confirmed in a similar report^75^. HI values for the adult age category were above the threshold limit, while HI values for children were within the tolerable limit. The sample study HI values were higher than the control sample values. The overall assessment showed that the borehole water samples are unfit for washing, bathing/showering. Adults are more prone to exposure to cancer health risks than children, which was in agreement with previous work^75^.

**Table 8:** ILCR values of average dermal dose.

PAHs	${\mathrm{ILCR}}_{\mathrm{Dermal}}$ (ADULT)		${\mathrm{ILCR}}_{\mathrm{Dermal}}$ (CHILD)	
	Study sample	Control sample	Study sample	Control sample
BbF	7.61 $\times {10}^{-5}$	7.46 $\times {10}^{-5}$	3.0 $\times {10}^{-5}$	2.34 $\times {10}^{-5}$
BaA	2.75 $\times {10}^{-5}$	8.22 $\times {10}^{-6}$	2.97 $\times {10}^{-6}$	2.2 $\times {10}^{-6}$
BaP	2.81 $\times {10}^{-5}$	1.83 $\times {10}^{-5}$	1.11 $\times {10}^{-5}$	7.22 $\times {10}^{-6}$
DBahA	7.85 $\times {10}^{-5}$	–	3.1 $\times {10}^{-6}$	–
HI	1.39 $\times {10}^{-4}$	1.01 $\times {10}^{-4}$	4.71 $\times {10}^{-5}$	3.28 $\times {10}^{-5}$

HQ and HI values for non-carcinogenic PAH exposure through the ingestion route are illustrated using Table 9. The hazard quotient values were < 1, which shows no chance of a non-carcinogenic effect^88,92,93^. The HI values for the study location were higher than the HI of the control location. The HI values were less than 1, which suggested no chance of contacting non-carcinogenic health risks.

**Table 9:** Hazard quotient for ingestion route.

n	${\mathrm{HQs }}_{\mathrm{Ingestion}}$ (ADULT)		${\mathrm{HQs }}_{\mathrm{Ingestion}}$ (CHILD)	
	Study Sample	Control Sample	Study Sample	Control Sample
Acp	0.0025	0.0103	0.0267	0.1098
Fla	0.0098	0.0068	0.1039	0.072
Flur	0.0015	0.0042	0.016	0.0448
Nap	0.0452	0.0098	0.4819	0.1045
HI	0.059	0.0311	0.6285	0.3311

The ILCR and HI values via the ingestion route are obtained in Table 10. The ILCR values were < 1E−04. The HI values were within the tolerable limit 1E−05. The adult HI was higher than the child HI, which showed that the adult has more chances of exposure to cancer risk through bioaccumulation ^75,86,90^.

**Table 10:** ILCR for ingestion route.

PAHs	${\mathrm{ILCR}}_{\mathrm{Ingestion}}$(ADULT)		${\mathrm{ILCR}}_{\mathrm{Ingestion}}$(CHILD)	
	Study sample	Control sample	Study sample	Control sample
BkF	2.25 $\times {10}^{-6}$	9.8 $\times {10}^{-6}$	1.12 $\times {10}^{-6}$	–
BbF	1.95 $\times {10}^{-5}$	5.85 $\times {10}^{-6}$	9.72 $\times {10}^{-6}$	7.6 $\times {10}^{-6}$
BaA	2.88 $\times {10}^{-6}$	1.27 $\times {10}^{-6}$	5.23 $\times {10}^{-6}$	1.1 $\times {10}^{-6}$
BaP	7.19 $\times {10}^{-6}$	3.13 $\times {10}^{-6}$	3.59 $\times {10}^{-5}$	2.3 $\times {10}^{-6}$
DBahA	9.4 $\times {10}^{-7}$	1.35 $\times {10}^{-8}$	4.68 $\times {10}^{-7}$	–
HI	3.28 $\times {10}^{-5}$	2.01 $\times {10}^{-5}$	2.01 $\times {10}^{-5}$	1.1 $\times {10}^{-5}$

## Conclusions

The borehole water samples were contaminated with PAHs through leachate runoff from rainfall. The total PAH concentration values obtained showed that the study sample was predominantly greater than the control sample due to its closeness to the dumpsite. The PAH levels in the borehole water samples were greater in the wet than in the dry season due to leachate infiltration from the dumpsite. The predominant ring in the study location was the 4–ringed PAHs, whereas the most dominant PAH group was the 5-ringed PAHs. The least dominant PAH group was the 6-ringed PAHs for both seasons and locations. The diagnostic ratios suggested both locations had mixed sources (petrogenic and pyrolytic). The TEQ and MEQ values were greater in the study samples than in the control samples. The individual PAH contributions to the TEQ and MEQ could trigger carcinogenic and non-carcinogenic health effects. Non-cancer risks seem unlikely for dermal contact and ingestion exposure routes. Carcinogenic risk through dermal contact exceeded the threshold limit for an adult and was lower for a child at the tolerable limit. Adults would be more susceptible to cancer risk than children. The HI values for carcinogenic risks through the ingestion pathway were within (1.0 $\times {10}^{-5}$) the acceptable limits for the adults and the children categories in all the locations. Based on the study's findings, there is a dire need to protect the environment and make it suitable for human lives by controlling the indiscriminate release of pollutants which often bioaccumulate to toxic levels if unmonitored. In addition, we recommend that the borehole water be treated before use to avoid health-related risks through domestic usage.

## Data Availability

The datasets used and/or analysed during the current study available from the corresponding author on reasonable request.
